# Psychiatric correlates of recurrent shoulder dislocations: the role of attention deficit hyperactivity disorder, impulsivity, and depression

**DOI:** 10.1515/med-2026-1471

**Published:** 2026-07-14

**Authors:** Sabri Kerem Diril, Orhun Celik, Yunus Elmas, Orhan Güneş, Hasan Gökçay, Soner Kocak, Cemil Ertürk

**Affiliations:** Department of Orthopaedics and Traumatology, Demiroglu Bilim University, Atasehir Florence Nightingale Hospital, Istanbul, Türkiye; Department of Orthopaedics and Traumatology, Goksun State Hospital, Kahramanmaraş, Türkiye; Department of Orthopaedics and Traumatology, Kanuni Sultan Suleyman Training and Research Hospital, Istanbul, Türkiye; Department of Orthopaedics and Traumatology, Tunceli State Hospital, Tunceli, Türkiye; Department of Psychiatry, Şarkışla State Hospital, Sivas, Türkiye

**Keywords:** shoulder dislocation, shoulder instability, arthroscopic bankart repair, impulsivity, attention deficit hyperactivity disorder, depression

## Abstract

**Objectives:**

One of the most common reasons for shoulder instability is recurrent shoulder instability. Arthroscopic repair is an option in the surgical treatment of Bankart lesions, and it is indicated if certain criteria are met. Recent evidence suggests that neurobehavioral traits may predispose individuals to orthopedic injuries. Therefore, we aimed to investigate the correlation between psychiatric parameters and recurrent dislocations in patients treated with arthroscopic Bankart repair.

**Methods:**

A total of 83 patients were included in the study, including 42 patients who underwent arthroscopic Bankart surgery due to recurrent shoulder dislocation (RSD) and 41 demographically matched healthy volunteers. The evaluated functional data included parameters such as Constant shoulder score and Rowe score for instability. Psychiatric parameters such as attention deficit hyperactivity disorder (ADHD), the Baratt impulsiveness Scale (BIS-11) and the Depression Anxiety and Stress Scale (DASS-21) were also evaluated.

**Results:**

The mean Constant shoulder score was 86.07 ± 12.07 (excellent) and the mean Rowe instability score was 81.26 ± 18.23 (good). The following psychiatric parameters were significantly greater in the patient group than in the control group.

**Conclusions:**

RSD patients were found to have significantly higher postoperative ADHD, BIS-11 and DASS-21 scores. Existing ADHD and impulsivity **may represent behavioral traits associated with** RSD. Higher DASS-21 scores in patients are associated with depression. We recommend that routine psychiatric evaluation for ADHD and impulsivity should be considered in the management of patients with RSD to improve outcomes and prevent recurrence.

## Introduction

Shoulder joint dislocations account for approximately 50 % of all major joint dislocations [1]. Up to 70 % of patients may experience recurrent instability after an initial dislocation [2]. Anterior dislocations often cause Bankart lesions – described as tearing of the labrum from the anteroinferior glenoid [3]. Arthroscopic Bankart repair is the most commonly performed surgical option and is indicated based on specific criteria [4]. Although many parameters have been investigated in the literature regarding the etiology of recurrent shoulder dislocation (RSD), few studies have evaluated psychiatric pathologies.

Although several physical and demographic risk factors for recurrent shoulder dislocation have been extensively investigated, the potential role of psychiatric and behavioral traits remains poorly understood [5], [6], [7], [8]. Given the known association between ADHD-related behavioral characteristics, impulsivity, and orthopedic injuries, we hypothesized that patients with recurrent shoulder dislocation would demonstrate higher ADHD, impulsivity, and psychological distress scores compared with healthy controls. Therefore, the aim of this study was to investigate the association between psychiatric parameters and recurrent shoulder dislocation in patients treated with arthroscopic Bankart repair.

## Methods

### Participants

Patients aged 18–60 years who underwent arthroscopic Bankart repair (2014–2022) with ≥1 year postoperative follow-up were included. All patients had a history of shoulder dislocations that had recurred more than 3 times. The patients were operated on with the indication of arthroscopic Bankart surgery due to complaints of instability.

### Exclusion criteria

– Prior to shoulder surgery, bony Bankart lesions were removed, or the Latarjet procedure was performed.
– Preexisting psychiatric/neurological disorders (to isolate the effect of undiagnosed traits).
– Generalized hyperlaxity (Beighton score >5/9).
– Major trauma or multitrauma patients.

### Assessments

– Functional outcomes: Constant shoulder score and Rowe instability score.
– Psychiatric measures:
  – ADHD symptoms (Adult ADHD Self-Report Scale).
  – Impulsivity (Barratt Impulsiveness Scale, BIS-11).
  – Depression/Anxiety Scale (DASS-21).
– Radiologic evaluation: X-ray, CT, MRI, and instability severity index (ISIS) score.

Surgically treated patients are called to the outpatient clinic for follow-up. At their last follow-up appointment, psychiatric, functional, demographic and clinical data were evaluated and recorded.

### Data collection and evaluation criteria

A total of 83 patients, consisting of 42 patients who were treated with arthroscopic Bankart repair and 41 healthy age- and sex-matched volunteer participants, were included in the study. **The control group consisted of age- and sex-matched healthy volunteers recruited from individuals presenting to the orthopedic outpatient clinic with non-shoulder-related complaints. Participants with a history of shoulder instability, shoulder trauma, previous shoulder surgery, diagnosed psychiatric disorders, or chronic neurological diseases were excluded from the control group.**

### Statistical analysis

While evaluating the findings obtained in the study, IBM SPSS Statistics 22 (IBM SPSS, Turkey) program was used for statistical analysis. While evaluating the study data, the conformity of the parameters to normal distribution was evaluated with Shapiro Wilks test. While evaluating the study data, in addition to descriptive statistical methods (Mean, Standard deviation, frequency), Mann Whitney U test was used in comparisons of parameters that did not show normal distribution between two groups in comparisons of quantitative data. Significance was evaluated at p<0.05 level.

### Study design and ethics

This retrospective case-control study was approved by the Institutional Ethics Committee of XXX Training and Research Hospital (Approval No: KAEK/2024.01.6). The study was conducted in accordance with the principles of the Declaration of Helsinki. Written informed consent was obtained from all participants, and all data were anonymized prior to analysis.

## Results

After the initial screening and application of the exclusion criteria, 42 patients who received arthroscopic Bankart repair due to RSD were eligible for inclusion in the study. Conversely, a control group of 41 age- and sex-matched healthy volunteers was included. Therefore, a total of 83 patients were included in the study. There were 42 patients (58.3 %) in the experimental group and 41 patients (41.7 %) in the control group. 30 (71.4 %) of the included patients were male, and 12 (28.6 %) were female in the experimental group. 28 male (68.3 %) patients and 13 female (31.7 %) patients were included in the control group. **The mean age was 33.9 ± 9.9 years in the experimental group and 35.8 ± 9.38 years in the control group, with no statistically significant difference between the groups (p=0.372).**

Constant shoulder score of the patients ranged between 59 and 100, with a mean value of **86.07 ± 12.07** (excellent) and a median value of 85, whereas the Rowe score of the patients ranged between 25 and 100, with a mean value of 81.26 ± 18.23 (good) and a median value of 80 (Table 1).

**Table 1:** Functional assessment of the patients in the experimental group.

Functional scores	Min–max	Mean ± SD (median)
Constant shoulder score	59–100	86.07 ± 12.07 (85)
Rowe score for instability	25–100	81.26 ± 18.23 (80)

SD, standard deviation; Min, minimum; Max, maximum.

The number of dislocations in patients ranged from 1 to 100, with a mean of 16.21 ± 21.63 and a median of 9.5. Sixty-one point nine percent of cases were operated on the right side and 38.1 % on the left side. 90 point five percent were right-sided dominant, while 9.5 % were left-sided dominant. The surgical dates are distributed as shown in Table 2. 61.9 % had 10 or fewer dislocations, while 38.1 % had more than 10 (Table 2).

**Table 2:** Demographic and clinical characteristics of patients in the experimental group.

		Min-max	Mean ± SD (median)
Number of dislocations, n=42		1–100	16.21 ± 21.63, 9.5
		n	%
Operated side, n=42	Right	26	61.9
	Left	16	38.1
Dominant side, n=42	Right	38	90.5
	Left	4	9.5
Surgical history, n=42	2012	1	2.4
	2014	6	14.3
	2015	3	7.1
	2016	4	9.5
	2018	3	7.1
	2019	4	9.5
	2020	5	11.9
	2021	13	31
	2022	3	7.1
Number of dislocations group, n=42	≤10	26	61.9
	>10	16	38.1

SD, standard deviation; Min, minimum; Max, maximum.

Psychiatric parameters were evaluated in the experimental and control groups via the Mann‒Whitney U test. ADHD had a mean value of 31.24 ± 10.94 and a median value of 30 in the experimental group, whereas it had a mean value of 17.49 ± 10.57 and a median value of 16 in the control group; these differences were highly significant (p<0.05). BIS-11 had a mean value of 66.71 ± 13.08 and a median value of 64 in the experimental group, whereas it had a mean value of 51.73 ± 7.26 and a median value of 52 in the control group, which was a highly significant difference (p<0.001). Stress scores had a mean value of 6.07 ± 3.71 and a median value of six in the experimental group, whereas it had a mean value of 4.51 ± 3.34 and a median value of four in the control group; these values were significantly different (p<0.05). Anxiety had a mean value of 6.05 ± 3.72 and a median value of 5 in the experimental group, whereas it had a mean value of 4.66 ± 2.9 and a median value of four in the control group; these values were not significantly different (p=0.078). The mean depression score was 5.52 ± 2.82, and the median score was six in the experimental group, whereas the mean score was 4.73 ± 3.36, and the median score was 5 in the control group; these values were not significantly different (p=0.242). **DASS-21 had a mean value of 17.64** ± **9.25 in the experimental group and 13.9** ± **8.9 in the control group, demonstrating a statistically significant difference between the groups (p=0.040)** (Table 3). **However, the depression and anxiety subscales did not demonstrate statistically significant differences between the groups.**

**Table 3:** Comparison of psychiatric parameters between the two groups.

	Patient group	Control group	p-Value
	Mean ± SD (median)	Mean ± SD (median)	
*ADHD*	31.24 ± 10.94 (30)	17.49 ± 10.57 (16)	0.001^a^
*BIS-11*	66.71 ± 13.08 (64)	51.73 ± 7.26 (52)	0.001^a^
Attention impulsivity	18.69 ± 4.85 (18)	12.41 ± 2.46 (12)	0.001^a^
Motor impulsivity	21.55 ± 5.16 (21)	15.51 ± 3.31 (16)	0.001^a^
Inability to plan	26.07 ± 6.91 (26)	23.8 ± 5.36 (23)	0.017^a^
*DASS-21*	17.64 ± 9.25 (15)	13.9 ± 8.9 (13)	0.040^a^
Stress	6.07 ± 3.71 (6)	4.51 ± 3.34 (4)	0.041^a^
Anxiety	6.05 ± 3.72 (5)	4.66 ± 2.9 (4)	0.078
Depression	5.52 ± 2.82 (6)	4.73 ± 3.36 (5)	0.242

SD, standard deviation, ^a^p<0.05.

A post-hoc power analysis was conducted using G*Power 3.1 to evaluate the statistical robustness of our findings. The analysis, configured for the Mann-Whitney U test with an alpha of 0.05 and sample sizes of 42 and 41, revealed a power (1-β) exceeding 99 % for the primary outcomes of ADHD (effect size d=1.07) and impulsivity, BIS-11 (d=1.14). For the DASS-21 score, a power of approximately 70 % was achieved for a moderate effect size (d=0.48).

## Discussion

**In the present study, we found that patients with recurrent shoulder dislocation treated with arthroscopic Bankart repair demonstrated significantly higher ADHD, BIS-11, and total DASS-21 scores compared with healthy controls. These findings suggest that certain behavioral and psychological traits may be associated with recurrent shoulder instability.** There are various studies that specifically portray the associations between psychiatric and orthopedic disorders, such as metacarpal fractures, voluntary shoulder dislocations, rotator cuff tears and adhesive capsulitis [5], [6], [7], [8], [9], [10], [11], [12]. We identified a specific behavioral trait among our surgically treated RSD patients. To the best of our knowledge, this study is the first of its kind. According to our study, patients with RSD who required surgical treatment presented significantly higher ADHD, BIS-11 and DASS-21 scores. The statistically significant difference in psychiatric scores suggests that psychiatric evaluation may be an important clinical factor in this population.

Many studies have emphasized various risk factors for RSD [13], [14], [15]. Among the well-known risk factors are age, sex, contact sports, competitive sports, overhead activities, family history and hyperlaxity [16], [17], [18], [19]. Balg et al. [1] defined the instability severity index score (ISIS) to distinguish patients eligible for arthroscopic repair from those who require open shoulder stabilization. They emphasized six main risk parameters: age, contact sports, competitive sports, hyperlaxity, Hill-Sachs lesion and glenoid appearance on X-ray. In addition, Tokish et al. [20] demonstrated the importance of parameters such as age, sportive activity, bone loss, type of instability, dominant side and sex in identifying surgical indications. In contrast, Kralinger et al. [21] concluded that the type of sports activity and RSD were not correlated. On the other hand, Longo et al. [22] reported a significant correlation between high-risk labour and RSD in a prospective study including 85 patients. However, they did not identify a significant correlation between RSD and parameters such as sex, dominant side, hyperlaxity, competitive sports, physical therapy after reduction of dislocation, return to sports, Hill-Sachs lesions and bony Bankart lesions. In a retrospective cohort study including 377 patients, Lebus et al. [23] emphasized the lack of consensus on predictors for surgical treatment of shoulder instability patients. They concluded that the utilization of the frequency, etiology, direction, and severity (FEDS) classification for shoulder instability may be effective. The current literature focuses on physical, social and morphological parameters associated with shoulder pathologies. However, the prevalence of preoperatively diagnosed psychiatric disorders among patients undergoing orthopedic sports medicine procedures is also high [24]. Therefore, our study aimed to evaluate RSD from a psychiatric perspective.

The literature has demonstrated that individuals with ADHD are at greater risk for orthopedic problems because of the behavioral traits associated with psychiatric conditions, such as impulsivity, aggression, motor coordination problems and risk-taking behaviors. In addition, some studies have linked ADHD symptoms to extremity fractures, burns, head trauma and self-inserting of nasal or aural foreign bodies [25], [26], [27], [28], [29]. In a systematic review by Polanczyk et al. [30], ADHD was defined as a chronic neurobehavioral disorder that has been common since a person’s childhood. In a retrospective study including 42 children by Erdogan et al. [31], ADHD was identified as a risk factor for first-time and recurrent bone fractures. In accordance with the current literature, which associates particular orthopedic conditions with ADHD, we observed that there was also a significant association between ADHD and patients with RSDs (p<0.001). Therefore, since ADHD is characterized by inattention, hyperactivity, and impulsivity, pre- and post-operative Adult ADHD Self-Report Scale (ASRS) assessment may be recommended for RSD patients. **Unlike postoperative psychological distress, ADHD is considered a chronic neurobehavioral disorder that typically begins in childhood; therefore, ADHD-related behavioral traits may more likely represent a preexisting vulnerability associated with recurrent shoulder instability rather than a consequence of the injury itself.**

The BIS-11 score is used to evaluate attentional impulsivity, motor impulsivity and nonplanning impulsivity. In a prospective study including 582 metacarpal fracture patients by Duramaz et al. [32], the BIS-11 score was significantly greater in the recurrent metacarpal fracture group than in the first-time metacarpal fracture and healthy groups (p<0.001). Furthermore, Kural et al. (7), in a comparative study of 30 patients with fifth metacarpal fractures, reported greater impulsivity of inability to plan in the patient group than in the control group. In addition, a study with 59 patients by Adıyeke et al. [33] reported that impulsivity scores were significantly higher in intentional fifth metacarpal fracture patients than in unintentional patients. In our study, the BIS-11 scores of the experimental group were significantly greater than those of the control group (p<0.001).

The DASS-21 is a scoring system that evaluates depression and anxiety. Many studies in the literature have analysed the relationship between shoulder pathologies and anxiety-depression. These studies reported that anxiety and depression levels due to shoulder pathologies are high. High anxiety and depression levels are also associated with poor outcomes (25, 26, 30). In the study reported by Lädermann et al. [34], shoulder instability was identified. affects the central nervous system, which causes emotional and cognitive changes as well as a high level of anxiety. Additionally, in a cross-sectional study including 594 acute hand/wrist fracture patients, Ross et al. [35] reported that pain and disability were significantly associated with depression and stress. **In our study, significantly higher total DASS-21 scores were identified in the patient group (p=0.040). However, depression and anxiety subscales were not significantly different between the groups. Therefore, these findings may reflect overall psychological distress rather than clinically significant depression or anxiety. Since psychiatric assessments were performed postoperatively, DASS-21 scores may also have been influenced by postoperative recovery status and previous dislocation experiences.** Nevertheless, it is difficult to conclude whether depression, stress and anxiety are components of RSD etiology or a result of this orthopedic condition.

In the current literature, many authors have identified a relationship between orthopedic pathologies and psychiatric disorders. **However, despite good functional shoulder and instability scores, patients demonstrated higher ADHD, impulsivity, and overall psychological distress scores compared with the control group**. **Therefore, considering psychiatric evaluation regarding the presence of ADHD, impulsivity, and psychological distress in patients presenting with RSD may be advisable to optimize patient management and achieve satisfactory outcomes. Recognition of ADHD symptoms and impulsive behavioral traits may help clinicians optimize postoperative rehabilitation protocols, improve patient compliance, and reduce risk-taking behaviors that may negatively affect shoulder stability and recovery outcomes.**

### Limitations

This study has several limitations. Our patient group consisted of RSDs treated with arthroscopic Bankart repair. We were able to identify RSD patients via a specific surgical method as a search parameter in the hospital database. However, there are RSD patients who are not treated surgically, who are treated in another facility or who are treated with different surgical methods. This might have caused unintentional selection bias.

Another limitation is the lack of preoperative psychiatric analysis of the patients. In our routine, no preoperative psychiatric examination is performed on any patient who does not have a psychiatric diagnosis. Patients in the present study demonstrated higher DASS-21 scores than did those in the control group despite good postoperative functional outcomes. However, DASS-21 scores may also have been low preoperatively for various reasons. **Additionally, psychiatric assessments were performed postoperatively, which may have influenced DASS-21 scores and limited interpretation of psychological distress findings**.

A comparison between single-time dislocators and RSD patients was not possible to further evaluate the relationship between shoulder dislocations and impulsivity. Additionally, **physical activity levels and sports participation were not systematically evaluated in either group, which may represent a potential confounding factor.**

A post-hoc power analysis confirmed that the study was sufficiently powered (>99 %) to detect the large effects of ADHD and impulsivity, which were the central focus of this investigation. However, the power for the DASS-21 outcome was lower (70 %), which is an expected issue in a single-center study. Therefore, further multicenter studies including more patients with preoperative as well as postoperative psychiatric analyses are necessary to elaborate this complex topic.

## Conclusions

Our aim was to evaluate the associations of certain psychiatric parameters with the RSD. To the best of our knowledge, this study is the first of its kind. The functional outcomes of the patients included in the study were good. However, RSD patients who were treated with arthroscopic Bankart repair were found to have significantly higher postoperative ADHD, BIS-11 and DASS-21 scores. Existing ADHD and impulsivity may be **associated** factors for RSD. Additionally, preoperative and postoperative psychiatric evaluations may be important for providing and maintaining satisfactory surgical results and avoiding further recurrence related to ADHD-related behavior. **Higher total DASS-21 scores may indicate increased psychological distress; however, depression and anxiety subscales were not significantly different between the groups.** This may be a preoperative occurrence **associated with** recurrent dislocations, considering good postoperative functional outcomes in the patient group. Therefore, **psychiatric evaluation may be helpful to identify psychological distress and behavioral traits that could influence postoperative rehabilitation and patient compliance**. Although the number of patients in this study was low, we believe that our study provides valuable scientific information. We recommend that routine psychiatric evaluation for ADHD and impulsivity should be considered in the management of patients with RSD to improve outcomes and prevent recurrence.
